# Cortical gray matter microstructural alterations in patients with type 2 diabetes mellitus

**DOI:** 10.1002/brb3.2746

**Published:** 2022-09-04

**Authors:** Haoming Huang, Xiaomeng Ma, Xiaomei Yue, Shangyu Kang, Yawen Rao, Wenjie Long, Yi Liang, Yifan Li, Yuna Chen, Wenjiao Lyu, Jinjian Wu, Xin Tan, Shijun Qiu

**Affiliations:** ^1^ The First Clinical Medical College Guangzhou University of Chinese Medicine Guangzhou Guangdong P.R. China; ^2^ Department of Radiology, The First Affiliated Hospital Guangzhou University of Chinese Medicine Guangzhou Guangdong P.R. China; ^3^ Department of Geriatrics, The First Affiliated Hospital Guangzhou University of Chinese Medicine Guangzhou Guangdong P.R. China

**Keywords:** diffusion tensor imaging, gray matter microstructural alterations, gray matter‐based spatial statistics, neurite orientation dispersion and density imaging, support vector machine, type 2 diabetes mellitus

## Abstract

**Background and purpose:**

Neurodegenerative processes are widespread in the brains of type 2 diabetes mellitus (T2DM) patients; gaps remain to exist in the current knowledge of the associated gray matter (GM) microstructural alterations.

**Methods:**

A cross‐sectional study was conducted to investigate alterations in GM microarchitecture in T2DM patients by diffusion tensor imaging and neurite orientation dispersion and density imaging (NODDI). Seventy‐eight T2DM patients and seventy‐four age‐, sex‐, and education level‐matched healthy controls (HCs) without cognitive impairment were recruited. Cortical macrostructure and GM microstructure were assessed by surface‐based analysis and GM‐based spatial statistics (GBSS), respectively. Machine learning models were trained to evaluate the diagnostic values of cortical intracellular volume fraction (ICVF) for the classification of T2DM versus HCs.

**Results:**

There were no differences in cortical thickness or area between the groups. GBSS analysis revealed similar GM microstructural patterns of significantly decreased fractional anisotropy, increased mean diffusivity and radial diffusivity in T2DM patients involving the frontal and parietal lobes, and significantly lower ICVF values were observed in nearly all brain regions of T2DM patients. A support vector machine model with a linear kernel was trained to realize the T2DM versus HC classification and exhibited the highest performance among the trained models, achieving an accuracy of 74% and an area under the curve of 83%.

**Conclusions:**

NODDI may help to probe the widespread GM neuritic density loss in T2DM patients occurs before measurable macrostructural alterations. The cortical ICVF values may provide valuable diagnostic information regarding the early GM microstructural alterations in T2DM.

## Graphical abstract

Detecting early cortical microstructural alterations in type 2 diabetes mellitus (T2DM) patients without cognition decline with gray matter‐based spatial statistics. Significantly lower intracellular volume fractions (ICVF) values were observed in nearly whole brain regions of T2DM patients. A linear support vector machine model was trained with cortical ICVF to realize the T2DM versus HC classification and exhibited the highest performance among the trained models, achieving an accuracy of 74% and an area under the curve of 83%.

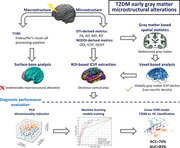

## 1 INTRODUCTION

Type 2 diabetes mellitus (T2DM) is a systematic metabolic disorder that affects approximately 415 million people worldwide and accounts for 90% of diabetes cases globally (Tripathi & Srivastava, 2006; Zheng et al., 2018). T2DM patients were at a 1.5‐2 times higher risk of neuropsychological dysfunction than healthy individuals (Cheng et al., 2012). Studies have revealed the association between accelerating brain aging related to T2DM and the development of cognitive impairment (CI) and Alzheimer's disease (Moran et al., 2019; Sato & Morishita, 2014). Neuroimaging evidence, especially in MRI studies, has shown alterations in the brains of T2DM patients.

T1‐weighted imaging (T1WI) helps identify macroscale abnormalities in the cortical gray matter (GM) region. A variable degree of brain atrophy associated with T2DM marked by loss of brain volume can be detected with T1WI (Geijselaers et al., 2015). A significant reduction in global brain volume has been found, along with atrophy in the orbitofrontal, hippocampus, basal ganglia, and occipital regions, in T2DM patients (Moulton et al., 2015). However, diffusion tensor imaging (DTI) is sensitive and objective for microscale pathologies and has long been used to study the changes in brain microstructure in many diseases (Drenthen et al., 2021; Scherfler et al., 2011; Yadav et al., 2020). Nonetheless, previous diffusion imaging studies mainly focused on white matter (WM) microstructural pathologies in T2DM patients, and the whole‐brain‐scale GM microstructure abnormalities underlying T2DM‐related brain damage are still unclear since DTI analysis of the GM is often susceptible to the partial volume effect of cerebrospinal fluid (CSF), which leads to deviations in the results (Henf et al., 2018).

The novel diffusion imaging reconstruction model neurite orientation dispersion and density imaging (NODDI) overcomes the shortcomings of DTI (Zhang et al., 2012). In this procedure, each voxel is divided into three microstructure intervals to provide corresponding metrics: (1) the intracellular volume fraction (ICVF), measuring the neuritic density; (2) the orientation dispersion index (ODI) reflecting the degree of neurite coherence; and (3) the isotropic volume fraction (ISOVF) representing the proportion of free water (Kamiya et al., 2020). Taking advantage of the multicompartment modeling of NODDI, a newly emerging method, named GM‐based spatial statistics (GBSS), was derived from tract‐based spatial statistics and can be used for unbiased analysis of GM microstructure in various diseases (Ball et al., 2013; Nazeri et al., 2017).

In this study, we analyzed the changes in the cortical GM of the brains of normal cognitive (NC) T2DM patients to identify the early cortical GM microstructural characteristics of diabetic brain damage. First, we analyzed cortical thickness and surface area with surface‐based analysis derived from T1WI. Subsequently, GBSS was used to investigate cortical GM microstructural alterations based on DTI and NODDI models, and the diagnostic value was further assessed by machine learning algorithms. Our research aimed to identify the changes in the cortical GM structure of the brains of T2DM patients and to provide new biomarkers for the detection of diabetes‐related brain damage.

## 2 METHODS

### 2.1 Participants

The current prospective cross‐sectional study was approved by the Medical Research Ethics Committee of the First Affiliated Hospital of Guangzhou University of Chinese Medicine (No. k[2020]115). All procedures performed in this study involving human participants followed the ethical standards of the institutional and/or national research committee and complied with the 1964 Helsinki Declaration and its later amendments or comparable ethical standards. Participants were recruited from among in‐hospital patients and clinic visitors admitted to the First Affiliated Hospital of Guangzhou University of Chinese Medicine from January to December 2021. Written informed consent was obtained from participants before the study. The diagnosis of T2DM was established based on guidelines from the American Diabetes Association recommendations (American Diabetes Association, 2019) (i.e., diabetes symptoms and hemoglobin A1c level (HbA1c) >6.5%, and/or a fasting blood glucose level (FBG) of > 7.0 mmol/L, and/or a random plasma glucose level of > 11.1 mmol/L and/or a 2‐h glucose level of >11.1 mmol/L after an oral glucose tolerance test). The healthy control (HC) participants were age‐, sex‐ and education level‐matched volunteers without any history of blood glucose abnormalities who underwent a routine physical examination, fasting finger‐prick blood glucose (GA‐6 blood glucose meter, Sinocare, China) and finger‐prick HbA1c (A1CNOW+ system, PTS Diagnostics, USA) to exclude potential diabetic subjects. All participants were right‐handed, Han Chinese adults. In addition, participants were examined in detail by experienced neurologists to ensure that they had no positive neurological symptoms.

For each registered participant, parameters such as age, sex, education level, systolic blood pressure, diastolic blood pressure, and body mass index (BMI) were recorded as basic information. All participants underwent a neuropsychological assessment, the Mini‐Mental State Examination (MMSE), to assess their cognitive status, and independent testers were blinded to the grouping information while performing the assessment. Plasma HbA1c (%), FBG, and fasting insulin (FIs, mU/L) levels were routinely tested for in‐hospital T2DM patients and were retrieved from the electronic medical records. Individual insulin‐resistant status was estimated by the homeostatic model assessment for insulin resistance (HOMA‐IR) which was calculated with HOMA2 Calculator 2.2.3 (OCDEM, Oxford, UK).

Subjects with the following characteristics were excluded from the trial: (1) participants with cognitive impairment (MMSE < 26); (2) participants with unstable blood glucose control; (3) organic lesions/abnormalities in the brain, such as tumors, infarction, hemorrhage, vascular malformation, head trauma, surgery, or congenital brain defects; (4) any previous history of neuropsychological diseases, such as epilepsy, depression, schizophrenia, or Parkinson's disease; (5) chronic infections, systemic diseases (e.g., organ failure and autoimmune diseases), a history of tumors, a history of alcohol dependence or substance abuse; (6) diabetes‐related complications (e.g., ketoacidosis, diabetic‐related retinopathy, nephropathy, or peripheral neuropathy); (7) moderate to severe hypertension (systolic blood pressure higher than 160 mmHg or diastolic blood pressure higher than 100 mm Hg), or hyperlipidemia; (8) contraindications to MRI examination, such as metallic implants or claustrophobia; (9) other types of abnormal glucose conditions or diabetes (e.g., impaired glucose tolerance, or type 1 diabetes mellitus); and (10) other factors that might affect thyroid function.

### 2.2 Magnetic resonance image acquisition and preprocessing

Images of all participants were acquired on a 3.0T MRI scanner (MAGNETOM Prisma, Siemens, Germany) equipped with a 64‐channel head coil. T2‐weighted imaging, T1WI, and T2‐fluid‐attenuated inversion recovery were acquired to rule out organic lesions/abnormalities, such as lacunar infarction and moderate to severe WM disease. Diffusion spectrum imaging and three‐dimensional T1WI were used for the analysis. The detailed MRI scanning parameters are shown in Table 1. Datasets with excessive head movement or poor image quality were excluded.

**TABLE 1:** MRI acquisition protocols

Sequence	TR/TE/TI (ms)	Slice thickness (mm) / gap	FOV (mm^2^)	Voxel size (mm^3^)	Acquisition time (min)
T1WI	2530/2.98/–	1/0	256 × 256	1 × 1 × 1	5:58
T2WI	3650/92/–	5/1	220 × 220	0.7 × 0.7 × 5	0:49
T2‐FLAIR	9000/84/2500	5/1	220 × 220	0.7 × 0.7 × 5	1:50
DSI*	4200/72/–	2/1	220 × 220	2 × 2 × 2	7:31

T1W1, T1‐weighted imaging; T2WI, T2‐weighted imaging; T2‐FLAIR, T2‐fluid‐attenuated inversion recovery; DSI, diffusion spectrum imaging; TR, repetition time; TE, echo time; TI, inversion time; FOV, field of view.

* Diffusion imaging data were acquired by using a half coverage Cartesian q‐space grid scheme with a radial grid size of 4. Eleven *b*‐values (*b* = 300, 350, 650, 950, 1000, 1350, 1650, 1700, 2000, 2700, and 3000 s/mm^2^) along 99 diffusion gradient directions were included in the acquisition, two *b* = 0 s/mm^2^ images were acquired, and one was taken in opposing phase encoding directions.

Diffusion data were first subjected to the *topup* tool in FSL 6.2.1 (FMRIB, Oxford, UK) for distortion correction. Subsequent preprocessing was performed with a 3D shoreline strategy in the Qsiprep package 0.14.3 (LINC, University of Pennsylvania, USA). The preprocessed diffusion data were reconstructed with the DTI model, and metrics, such as fractional anisotropy (FA), mean diffusivity (MD), axial diffusivity (AD), and radial diffusivity (RD), were then calculated with the *dtifit* command in FSL. The AMICO toolkit 1.2.10 (Department of Computer Science, University of Verona, Italy) was used to reconstruct the NODDI model with associated metrics (ICVF, ODI, and ISOVF).

### 2.3 Surface‐based analysis

T1WI data were imported into FreeSurfer 7.2.0 (MGH, Harvard Medical School, USA) for the automatic recon‐all processing pipeline. Gaussian kernel smoothing with a full width at half a maximum of 15 mm was chosen for all surface maps. Additionally, the estimated total intracranial volume (eTIV) for each subject was calculated.

### 2.4 GBSS

GM microstructure was assessed using a GM‐based spatial statistics approach on DTI‐ and NODDI‐derived metrics as described in a previous study (Nazeri et al., 2017) with slight modifications to fit the current study. The population‐based GM was skeletonized to preserve only the voxels with a GM fraction > 0.65 in 70% of the participants. Diffusion metric maps were then projected onto the GM skeleton with the maximum GM fraction.

### 2.5 Statistical analysis

Statistical tests were performed in R 4.2 (R Core Team and R Foundation). Data analysts were blinded to the grouping labels in the data analysis procedures. Sex distribution among the two groups was compared through the chi‐square test. Significant differences between two groups in other demographic, clinical, and psychological variables were identified by the *t‐*test if data were normally distributed; otherwise, the Kruskal–Wallis test was used. Statistical tests were performed at a significance level set to .05.

Cortical thickness and area were assessed with the vertex‐based general linear model, which fitted with the cortical thickness or area as a dependent factor and group as the independent variable to which age, sex, education level, BMI, and eTIV were regressed out as covariates. Vertices above the thresholding value of 3.0 were considered part of clusters that were tested for statistical significance. The resulting maps were further corrected for the multicomparison of both hemispheres with a clusterwise *p* threshold of .001.

Population‐space GM skeleton mappings were subjected to FSL's *randomise* program for voxelwise analyses between two groups using a nonparametric permutation test with 10,000 permutations and adjusting for age, sex, education level, BMI, and eTIV with familywise error (FWE, *p_fwe_* < .05) correction, followed by threshold‐free cluster enhancement (TFCE), resulting in 1 – *p* mappings for diffusion metrics. Significantly different GM regions were clustered with the *cluster* command in FSL with cluster sizes >100 voxels and a 1 – *p* threshold of .95.

Finally, we explored the diagnostic accuracy of the NODDI metric ICVF for T2DM and healthy subjects. To structurally analyze the metric, the Destrieux cortical deterministic atlas (Destrieux et al., 2010) was nonlinearly registered to the subjects’ space, and the mean ICVF values of regions of interest (ROIs) were extracted from the subjects. Data randomly were split into the training and the test set in a 7:3 ratio. Principal component analysis was used for dimensionality reduction, and components that explained 99% of the variance in the training set were selected. Four machine learning algorithms were tested for the classification task, namely, K‐nearest neighbors (KNN), logistic regression classification (LRC), and the linear kernel support vector machine (SVM) and radial basis function (RBF) kernel SVM. In the training process, a grid search strategy was performed for parameter optimization with fivefold cross‐validation using scikit‐learn 1.1.1. The performances of the algorithms were evaluated with weighted average precision, recall, accuracy rate, and *F*1‐score. For each algorithm, a receiver operating characteristic (ROC) and the area under the curve (AUC) were also generated.

## 3 RESULTS

### 3.1 Participants’ characteristics

**FIGURE 1:**
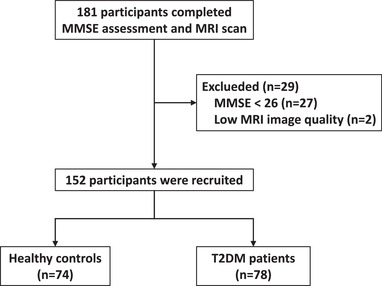
Study flowchart. T2DM, type 2 diabetes mellitus; MMSE, Mini‐Mental State Examination

In total, 181 participants completed the MRI scans and all the assessments; 27 participants were excluded due to CI (MMSE <26), and 2 participants were excluded due to poor image quality (Figure 1). Seventy‐eight T2DM patients and 74 HCs were finally enrolled in the current study. The demographic, clinical, and cognitive measurements are summarized in Table 2. No significant differences in sex, age, or education levels between the participants in the two groups were observed. The average diabetes duration in the T2DM group was 4.08 ± 3.58 years. Compared to the HC group, HbA1c and fasting glucose levels were significantly higher in the T2DM group (*p* < .001). Regarding clinical testing and psychological cognitive assessment, BMI, MMSE scores, the systolic and diastolic blood pressure also showed no significant differences between groups (*p* > .05).

**TABLE 2:** Demographic and clinical information and neuropsychological assessment results of participants in the two groups

	HC	T2DM	Statistics	*p*
Groups	(*n* = 74)	(*n* = 78)	*χ* ^2^/*t*	Value
Age (years)	47.82 (10.31)	46.96 (9.56)	−0.534	.593
Sex = male (%)	26 (35.1)	29 (37.2)	0.009	.926
Education level (years)	10.98 (3.42)	11.33 (3.77)	0.591	.563
Systolic blood pressure (mmHg)	125.31 (17.28)	128.89 (16.76)	1.294	.197
Diastolic blood pressure (mmHg)	83.06 (10.98)	85.11 (9.25)	1.243	.214
BMI	23.20 (3.08)	24.08 (3.31)	1.690	.093
Diabetes duration (years)	–	4.08 (3.58)		–
HbA1c (%)*	5.24 (0.76)	9.10 (2.57)	12.680	<.001^†^
Fasting glucose level (mmol/L)*	4.84 (0.63)	8.85 (2.92)	11.816	<.001^†^
Fasting insulin (mU/L)	–	12.13 (11.12)		–
HOMA‐IR	–	1.74 (1.50)		–
MMSE	29.50 [28.00, 30.00]	29.00 [28.00, 30.00]	3.026	.082
eTIV (cm^3^)	1496.47 (154.29)	1534.65 (139.02)	1.600	.111

*Note*: The data are expressed as the mean and standard deviation when the data are normally distributed; otherwise, they are expressed as the median and 25% and 75% interquartile range.

T2DM, type 2 diabetes mellitus; HC, healthy control; BMI, body mass index; HbA1c, glycosylated hemoglobin A1c level; HOMA‐IR, homeostatic model assessment for insulin resistance; MMSE, Mini‐Mental State Examination; eTIV, estimated total intracranial volume.

* Plasma gluc tests in the T2DM group and fasting finger‐prick blood tests in the HC group.

^†^ *p* < 0.05.

### 3.2 Cortical thickness and area

In the surface‐based analysis, the cortical area and thickness of participants were studied. No significant group difference in T2DM and HC participants was detected after a cluster thresholding value of 3.0.

### 3.3 Microstructural aberrants in the GM

**FIGURE 2:**
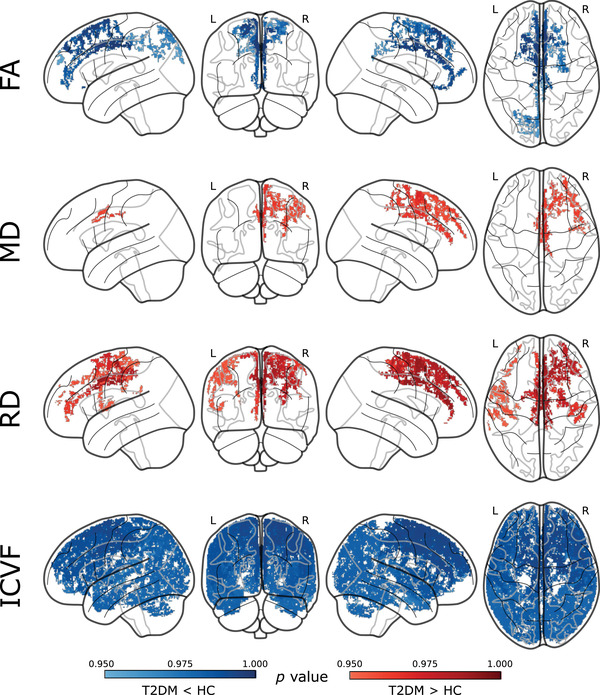
Patterns of the altered microstructure of the GM between participants with T2DM and HCs (FEW corrected with TFCE). T2DM, type 2 diabetes mellitus; HC, healthy control; FA, fractional anisotropy; MD, mean diffusivity; RD, radial diffusivity; ISOVF, isotropic volume fraction; color bar represents the (1 – *p*) values; red color indicates significantly increased metric value in T2DM group with (1 – *p*) > .95; Blue color indicates significantly decreased metric value in T2DM group with (1 – *p*) > .95

GBSS analysis was performed to study the pattern of the altered microstructure in the GM between participants with T2DM and HCs (Table 3 and Figure 2). We observed a significant decline in FA across the frontal, parietal, and occipital regions in the T2DM group. Higher MD values were observed across the frontal and parietal lobes in the T2DM group. RD values were also increased in the T2DM group and exhibited similar patterns with frontal, parietal, insular, and temporal region involvement. Surprisingly, we found nearly the whole brain GM region with decreased ICVF in T2DM patients compared with HC participants, predominantly in the frontal, parietal, temporal, occipital, cerebellum, and insular regions, as well as some subcortical and cerebellar structures. No GM regions had significantly higher FA and ICVF values and lower MD and RD values in the participants in the T2DM group compared to the HCs. Furthermore, there were no significant GM differences in the AD and ODI values between the T2DM group and HC group.

**TABLE 3:** Clusters with significantly different diffusion metrics in the T2DM group compared to the HC group

				MNI coordinates of peak voxel			
Diffusionmetric	Cluster Index	Voxels	Peak (1 – *p*)	*X*	*Y*	*Z*	Anatomical region(% of all clusters overlapped)*
FA (T2DM < HC)	1	7423	0.998	–2	–9	41	Frontal lobe (61.21) Parietal lobe (13.07) Occipital lobe (2.36)
	2	1091	0.979	–14	–74	56	
	3	407	0.965	–11	–88	39	
	4	114	0.962	–7	58	35	
MD (T2DM > HC)	1	1614	0.972	5	5	60	Frontal lobe (73.36) Parietal lobe (3.09)
	2	1498	0.965	13	27	61	
	3	340	0.965	43	18	52	
	4	217	0.962	–5	3	44	
	5	191	0.959	44	–2	58	
RD (T2DM > HC)	1	8150	0.992	4	10	37	Frontal lobe (63.79) Parietal lobe (6.53) Insula (0.03) Temporal lobe (0.01)
	2	666	0.960	–42	–20	56	
	3	252	0.957	–58	10	21	
	4	229	0.961	–57	–18	45	
	5	200	0.957	–47	14	45	
	6	156	0.956	–44	–5	11	
	7	153	0.957	–49	7	38	
	8	142	0.961	–34	40	37	
	9	133	0.955	–38	32	43	
	10	104	0.953	–46	–36	61	
ICVF (T2DM < HC)	1	76226	0.993	28	–1	64	Frontal lobe (26.60) Parietal lobe (15.29) Temporal lobe (14.03) Occipital lobe (9.35) Cerebellum (4.30) Insula (1.72)

T2DM, type 2 diabetes mellitus; HC, healthy control; FA, fractional anisotropy; MD, mean diffusivity; RD, radial diffusivity; ISOVF, isotropic volume fraction.

* The percentage of the total volume of all the clusters that overlapped with the structure.

### 3.4 Classification of T2DM versus HCs with cortical ICVF

**FIGURE 3:**
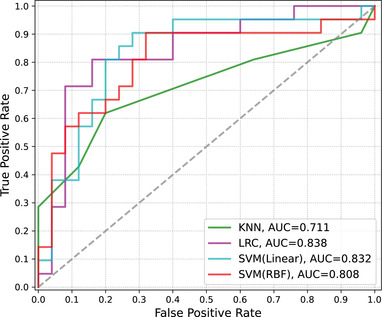
Receiver operating characteristic curves of the trained models for the classification of T2DM versus HCs. KNN, K‐nearest neighbors; LRC, logistic regression classification; SVM, support vector machine; Linear, SVM with the linear kernel; RBF, SVM with radial basis function kernel; AUC, area under the curve

In the GBSS analysis, nearly whole ICVF decremental abnormalities were observed in T2DM patients. To assess the diagnostic value of ICVF for T2DM brain damage, the Destrieux cortical deterministic atlas was adopted and nonlinearly registered to the subject's diffusion space. A total of 150 ROIs (cortical structures) were defined in the Destrieux atlas (75 per hemisphere), and the mean IVCF for each subject was extracted from the ROIs. All 152 subjects were randomly split into a training set (53 T2DM patients and 53 HCs) and a validation set (25 T2DM patients and 21 HCs) with diabetic status as the label. PCA was subsequently performed for dimensionality reduction, and components that explained 99% of the variance in the training set were selected. Data were transformed with the PCA model before further analysis. Four ML algorithms were tested for classification with a grid search strategy and fivefold cross validation in the model training (Table 4). In the KNN model, the optimized number of neighbors was 10, and the model showed a precision rate of 72%, a recall rate of 72%, and an *F*1‐score of 71%. The accuracy for KNN was 72%. The *L*2 penalty was used in LRC model training, and the optimal inverse of regularization strength (*C* values) was 37.93. The LRC model showed a precision rate of 74%, a recall rate of 67%, and an *F*1‐score of 66%. The accuracy for the LRC was 67%. In the SVM model with a linear kernel, the optimal *C* value was 61.58, and the model yielded a precision rate of 80%, a recall rate of 74%, an *F*1 score of 73%, and an accuracy of 74%. An additional SVM model with an RBF kernel was also trained with an optimal *C* value of 5.46 and an inverse of the radius of 2.07, showing a precision rate of 72%, a recall rate of 65%, an *F*1‐score of 64%, and an accuracy of 65%. ROC curves were generated (Figure 3), with AUCs for the KNN, LRC, SVM with a linear kernel, and SVM with an RBF kernel models of 0.711, 0.838, 0.832, and 0.808, respectively.

**TABLE 4:** Performance of the trained models for the classification of T2DM versus HCs

	Weighted avg.	Weighted avg.	Weighted avg.		
Model	precision	recall	*F*1‐score	ACC	AUC
KNN	0.72	0.72	0.71	0.72	0.72
LRC	0.74	0.69	0.67	0.67	0.84
SVM (linear )	0.80	0.74	0.73	0.74	0.83
SVM (RBF kernel)	0.72	0.65	0.64	0.65	0.81

Avg., average; KNN, K‐nearest neighbors; LRC, logistic regression classification; SVM, support vector machine; Linear, SVM with the linear kernel; RBF, radial basis function; ACC, accuracy; AUC, the area under the curve.

## 4 DISCUSSION

In the current study, we investigated the cortical macrostructural and microstructural alterations in T2DM patients without cognitive decline. T2DM affects brain structure, although the mechanisms underlying these effects are unclear. Previous studies indicated that T2DM patients with peripheral neuropathy showed changes in the cortical thickness of the corresponding brain regions (Hajek et al., 2016; Zhang et al., 2020); moreover, T2DM patients with MCI showed bidirectional changes in cortical thickness in multiple brain regions (Li et al., 2018). Nonetheless, a 5‐year longitudinal study did not find that T2DM had a direct effect on cortical thickness or cognitive decline, but there are ways to indirectly link T2DM and cognitive decline through baseline cortical thickness (Moran et al., 2019). In the surface‐based analysis, our results indicated that compared to the values of HC subjects, the cortical thickness and surface area values of T2DM subjects did not change significantly. Our results are consistent with those of a previous longitudinal study; the diabetes durations of the T2DM participants were relatively short, and the damage to brain tissue may not yet be reflected in the macrostructure.

Since no different macroscale changes were detected, we applied the GBSS method to quantitatively evaluate GM microstructure changes in T2DM. In the GBSS analysis, AD derived from the DTI model was not significantly different between the groups, indicating that cell integrity was intact in T2DM. In addition, the ODI and ISOVF derived from the NODDI model did not reveal GM areas with abnormalities, which means that there was no significant change in the orientation dispersion between the participants in the two groups. We found that diabetic patients mainly exhibited a reduction in the FA and increments in the MD and RD of the bilateral frontal and parietal cortices, which means neurodegeneration was significant in the above regions. Moreover, GBSS analysis highlighted significantly decreased ICVF involving nearly the whole brain GM region of T2DM patients, indicating density loss of neurites in the brain microarchitecture (Kara et al., 2014).

The use of the DTI and NODDI has been reported in a previous study on brain WM microstructural alterations in T2DM patients, identified larger WM regions showed decreased FA and ICVF in T2DM patients with MCI, and the ICVF values in the genu of the corpus callosum and thalamic regions correlated with the HbA1c level, diabetes duration, and cognitive performance (Xiong et al., 2019). DTI‐derived metrics were used as quantitative indicators of brain WM microstructural alterations in diseases (Andica et al., 2020; Dennis & Thompson, 2013). Nonetheless, the DTI model, assuming a Gaussian distribution of water molecules (Andersson et al., 2017), may not be suitable for specifically identifying the microstructural characteristics in the GM that consist of neurites. NODDI based on the multicompartment biophysical model can effectively preclude the influence of the partial volume effect caused by the CSF (Assaf et al., 2013). With the advantages of the NODDI model, GBSS analysis tests for GM microstructural alterations on a whole‐brain scale with a voxelwise approach, allowing the detection of density and orientation changes in GM neurite structure (Fukutomi et al., 2018; Vogt et al., 2020), which has attracted interest in a variety of psychiatric and neurological conditions (Kamagata et al., 2017; Nazeri et al., 2017).

In our study, we applied GBSS with DTI and NODDI metrics. Although similar patterns of GM microstructural alterations were observed with FA, MD, and RD metrics in T2DM patients across frontal and parietal regions, a much larger area of microstructural abnormalities in T2DM patients was reflected with ICVF. In ex vivo and in vivo studies (Fukutomi et al., 2018; Grussu et al., 2017), evidence showed that ICVF reflects myelinated axonal density in GM and ODI reflects the variation of the myelinated axonal orientation, which may capture the processes during brain damage preceding neurite loss in GM (Fukutomi et al., 2018). Moreover, dispersion of the orientation and density of neurites are the two key contributors to FA (Jeurissen et al., 2013; Vos et al., 2012); hence, NODDI may be advantageous for capturing the specific microstructural characteristics of neurites in GM. Previous studies demonstrated peripheral axonal dysfunction and myelinated axonal diameter reduction in T2DM patients (Kwai et al., 2016; Mohseni et al., 2017). However, gaps exist in the current knowledge of GM microstructural alterations in T2DM. The findings of this study indicated that widespread GM myelinated axonal density loss in T2DM patients may be an early characteristic before macrostructural changes.

To explore the diagnostic value of neuritic density loss for T2DM versus HCs, the mean ICVFs were extracted from subjects with ROIs defined in the Destrieux cortical deterministic atlas. We trained four frequently used machine learning models for the classification task, namely KNN, LRC SVM with linear kernel, and SVM with RBF kernel, and the SVM model with linear kernel exhibited the best performance among the train models and the KNN performed worst. Because using a low dimensional feature space in classification tasks can improve the classification efficiency and mitigate the overfitting problem (Jin et al., 2019), we used PCA for dimensionality reduction. After PCA transformation, the SVM with linear kernel achieved accuracy and AUC of 74% and 0.83, respectively in distinguishing T2DM patients from HCs. LRC showed higher AUC than that of linear kernel SVM, but the accuracy of the LRC model was 67% compared to 74% in SVM. SVM shows good overall performances in classification tasks of neuroimaging and is the most popular machine learning algorithm in neuroimaging studies (Arbabshirani et al., 2017; Yassin et al., 2020). As showed in GBSS analysis, the GM ICVF was unidirectional altered (decreased) in T2DM, linear kernel SVM model may be more capable of capturing this type of linear pattern, despite nonlinear kernels for SVM models, such as polynomial and RBF kernel, have more hyperparameters and are more capable to learn nonlinear patterns behind the neuroimaging data (Arbabshirani et al., 2017). Machine learning and deep learning methods have been introduced to discriminate T2DM and cognitive conditions from structural and functional MRI. Our recent study showed that deep transfer learning in T1WI data achieves accuracies of 60.48% and 62.90% for identifying T2DM‐CI versus T2DM normal cognition (NC) and T2DM versus HCs, respectively (Chen et al., 2021). Higher classification performances were also reported in studies on single‐digit brain function connections and resting‐state functional connections with SVM and E‐net models in the detection of T2DM and cognitive conditions (Liu et al., 2019; Qian et al., 2020). Although our model exhibited a moderate performance compared to previous research, the findings suggested that simple ICVF values extracted from subjects can provide valuable diagnostic information and be used for clinical implementation regarding GM microstructural alterations, while macrostructural damage is still silent in T2DM.

There are some limitations in the present study. First, the current study used diffusion maps to generate the GM skeleton with a well‐developed GBSS pipeline, while a study argued that processing low‐resolution diffusion images is challenging due to partial volume effects and artifacts. The incorporation of spatial surface‐based maps and NODDI may exhibit higher sensitivity in capturing differences (Parvathaneni et al., 2019). Second, we focused on microstructural changes in this study, and future functional MRI studies can be conducted to investigate the synchronized functional alterations in GM regions that are significantly altered in T2DM patients. Third, cognitive dysfunction is the major clinical concern following T2DM brain damage, and the present study only focused on the microstructural alterations in T2DM without CI. Fourth, although GBSS analysis revealed some subcortical and cerebellar GM microstructural alterations, the current study focused on cortical microstructural abnormalities in T2DM patients. Fifth, the diagnostic value results of cortical ICVF are exploratory, further external validity is a need in future studies. Sixth, the current study was a single‐center observational study, and selection bias was not inevitable. Finally, the healthy subjects were recruited based on self‐report medical history and fasting finger‐prick blood tests, which are not sufficient to rule out glucose metabolization disorders. Further research is warranted to investigate cognitive dysfunction‐specific GM characteristics and to elucidate the underlying mechanisms that produce the structural and microstructural neuroimaging findings in patients with T2DM.

## 5 CONCLUSIONS

In conclusion, diabetic neuropathologies have accumulated over the years, and widespread GM neuritic density loss in T2DM patients may be an early characteristic before overt macrostructural changes. The findings also suggest that NODDI metrics provide valuable knowledge regarding GM neurodegenerations that are not detectable using T1WI or DTI, and cortical ICVF values may be used for clinical implementation in distinguishing early T2DM brain damage. Our study furthers the understanding of the early neuropathologies in T2DM.

## CONSENT TO PARTICIPATE

The study adhered to the Declarations of Helsinki. All participants provided written informed consent.

## AUTHOR CONTRIBUTIONS

XMM, HMH, WJL, and XMY wrote the manuscript. WJLyu, JJW, YNC, and SYK were responsible for recruiting subjects. XMM, XMY, YL, and YFL collected multimodal MRI data. YL, XT was responsible for the clinical MRI report. JJW, WJLyu, SYK, and YWR collected psychological test data. XMM and HMH analyzed the data. XT, HMH, and SJQ designed and coordinated the study. All authors read and approved the final manuscript.

## COMPETING INTERESTS

The authors declare that they have no conflict of interest.

### PEER REVIEW

The peer review history for this article is available at: https://publons.com/publon/10.1002/brb3.2746.

## Data Availability

The data sets used and/or analyzed during the current study are available from the corresponding author on reasonable request.
